# Tissue Engineering Supporting Regenerative Strategies to Enhance Clinical Orthodontics and Dentofacial Orthopaedics: A Scoping, Perspective Review

**DOI:** 10.3390/biomedicines11030795

**Published:** 2023-03-06

**Authors:** Mushriq Abid, Hasan Jamal, Elham Alsahafi, Arkadiusz Dziedzic, Robert Kubina

**Affiliations:** 1 Department of Orthodontics, College of Dentistry, University of Baghdad, Baghdad 01110, Iraq; 2 Independent Researcher, Makkah 24371, Saudi Arabia; 3 Department of Basic and Clinical Sciences, Faculty of Dentistry, Umm AlQura University, Makkah 21955, Saudi Arabia; 4 Department of Conservative Dentistry with Endodontics, Medical University of Silesia, 40-055 Katowice, Poland; 5 Department of Pathology, Medical University of Silesia, 40-055 Katowice, Poland; 6 Silesia LabMed, Centre or Research and Implementation, Medical University of Silesia, 40-752 Katowice, Poland

**Keywords:** dental medicine, tissue regeneration, stem cells, orthodontics, dentofacial orthopedics, orthognathic surgery, tissue engineering

## Abstract

The personalized regenerative therapeutic strategies applicable in the structural and functional repair of maxillofacial/dental defects are expected to extend beyond the limits of what is currently possible in the management of dentofacial anomalies and treating malocclusions. The application of undifferentiated stem cells (SCs), including signaling molecule control and individualized tissue engineering based on targeted therapies, has been proposed to overcome therapeutic limitations and complications associated with treatments for craniofacial defects, including severe orthodontic discrepancies. This scoping, prospective review discusses comprehensively the current knowledge and prospects for improving clinical outcomes by the application of novel cell-required and cell-free regenerative strategies in biomedicine. The existing evidence, although scant, suggests that patients receiving an orthodontic treatment could benefit from precise tissue augmentation, allowing enhancement of tooth movement generated by orthognathic forces; faster, more predictable alignment of dental arches; optimal management of periodontal complications; and prevention of external root resorption. Ultimately, enriching orofacial tissues and “customizing” the repair of congenital/acquired defects in the craniofacial region can be vastly enhanced to provide a positive therapeutic outcome and improve patients’ quality of life.

## 1. Introduction

An emerging interdisciplinary field, regenerative dentofacial medicine (RDM) focuses on the development and application of treatments to establish and/or restore normal function and appearance of dentition and related structures [1]. Significant research efforts over the last decades have contributed to understanding the development and function of dental, oral, and dentofacial hard and soft tissues, as well as the biology of healing and the processes of oral/dentofacial tissue regeneration/repair [2,3]. Whilst there has been reported a relatively high prevalence of dental malocclusion/dentofacial malformations ranged 22.5–93% globally [4], as new generations of treatment modalities mimicking biological processes, as well as biomaterials for tissue augmentation have proliferated, therapeutic protocols have been refined to incorporate biological principles and a minimally invasive approach in dentofacial orthopedics (DFO) and routine orthodontics. The innovative RDM methods were targeted especially at maxillofacial defects (cleft and lip palate), acceleration of orthodontic tooth movement, and prevention of periodontal complications during orthodontic therapy. Various treatment modalities based on RDB have been described, investigated in pre-clinical settings, and proposed for clinical application in orthognathic management, including the incorporation of 3D biomimetic scaffolds (BSs) [5,6], advances in oral stem cells for tissue regeneration [4,7,8,9], biomaterials [10,11], growth factors (GFs) [7,12,13,14], and tissue-engineered pre-vascularized bone and soft tissue flaps. The complex intercellular mechanism, cells’ interplay, signaling pathways, and molecules determine the optimal regeneration of vital elements during the induced tissue-engineered reconstruction of oral and dentofacial structures.

This brief scoping literature review discusses the up-to-date status of the field of regenerative dental medicine, considering a potential application of primarily stem cell-based methods, specifically in clinical orthodontics and dentofacial orthopedics. A comprehensive review with a narrative approach has been designed to provide an impetus for further research on the personalized, regenerative therapies utilizing tissue-engineered methods in an orthodontic practice and complex cases of dentofacial orthopedics.

Initially, the simplified online search strategy of existing in vitro and clinical study results was applied by exploring the main electronic databases, utilizing a semi-structured, qualitative approach restricted to English-language articles only, and studies published since 2000. Following a preliminary assessment of the scant evidence for existing orthodontic-related regenerative therapy, a structured systematic review could not be conducted due to insufficient data specific to dentofacial orthopedics, a substantial heterogeneity of studies, a lack of a clear research question, and the potential bias of studies [15,16]. The broad range of pre-defined eligibility criteria was allocated because of the multidisciplinary and multifaceted, complex profile of “regenerative medicine/dentistry” field, involving also invasive interventions associated with oral/maxillofacial surgery. Intentionally, to expand a range of eligible sources, all available studies were taken into consideration, including in vitro, animal-based, clinical observational/experimental studies, as well as systematic reviews, umbrella reviews, case reports, and clinical trials.

## 2. Stem Cell-Based Regenerative Strategies in Orthognathic Dentistry

Various stem cells (SCs) have been widely investigated in vitro and in vivo on animal models for potential applications in dental medicine for the past two decades. SCs are defined as undifferentiated cells that can give rise to different specialized cell types or self-renew under suitable conditions [8,9,17]. Apart from well-studied mesenchymal stem cells (MSCs) from bone marrow, numerous sources of adult stem cells have been investigated, such as umbilical cord blood, muscle, adipose tissue, and teeth [18,19]. SCs derived from non-dental tissues have also been investigated; however, oral and dental sources are deemed more suitable for clinical application because of their developmental origin (Table 1).

Adult human dental pulp stem cells were first discovered in the 21st century when researchers isolated self-renewing cells capable of forming several different cell lineages in vitro [7,8,9,17,18]. To date, a total of five stem/progenitor cells from human dental tissue have been identified (Table 2).

Different types of oral SCs can be potentially utilized in clinical orthodontics and DFO. Primarily, dental pulp SCs have been identified as a promising source of stem cells for the treatment of medical conditions by numerous research teams to date, but these cells may not be easily accessible. Clinical orthodontics as well as oral surgery are two fields in which dental pulp SCs can be obtained since the extraction of sound, intact primary or permanent teeth, usually, premolars and selected molars, is a common approach for treating malocclusion. Subsequent storage of extracted teeth within tooth banking facilities allows isolation of SCs, culturing in the laboratory, and cryopreservation for future use. However, as tissue banking is associated with advanced biotechnological facilities and high costs of SCs acquisition, this novel modality is not currently covered by national public health and insurance schemes since limited evidence exists confirming the usefulness of cryopreserved cells for clinical applications [20,21].

The laborious good manufacturing practice (GMP)-compliant production of cell therapeutics could be streamlined by the adoption of automated cell processing platforms, which are already used in the hematology field. The main disadvantage is associated with significant cost and advanced equipment. It has recently been suggested that SCs grown in microgravity conditions may have improved clinical properties and that large-scale production of stem cell-derived products could benefit from manufacturing in a microgravity environment [22]. For carefully selected clinical applications, an enhanced therapeutic outcome can be achieved using autogenous, minimally processed tissue derivatives. The most recent randomized control trial reported on applying autogenous micrografts containing freshly harvested dental pulp that had been mechanically dissociated using a short chairside protocol in periodontal regenerative surgery [23]. A similar protocol seems adequate to be used in some orthognathic surgeries. Interestingly, both in vitro and in vivo studies suggest that even inflamed and infected dental and oral tissues could be useful sources of cells for regenerative therapies; for example, inflamed dental pulp and periodontal granulation tissue [24,25,26].

## 3. Scaffolding-Based, Cell-Free Regenerative Strategies on Maxillofacial and Orthognathic Surgery

Besides well-described procedures utilizing various types of stem cells, cell-free strategies, including a wide range of growth factors and 3D scaffolds, have been investigated in the regeneration of craniofacial bony defects [27,28,29], the details of which are beyond the scope of this review.

Recently, extracellular vesicles have been explored for use in regenerative periodontics [30,31,32,33,34]. Because of their epigenetic capacity and microRNA cargoes, MSC-derived extracellular vesicles (EVs), termed exosomes, containing physiologically active molecules such as GFs, cytokines, and microRNAs have attracted special interest in cell-free regenerative treatment [35,36,37]. The emerging discovery of extracellular microRNAs in gingival crevicular fluid (GCF) has shed light on their potential for use in the modification of orthodontic tooth movement [38]. The exosome originates from the endosome and has a size range of 30–100 nm [20]. Exosomes provide benefits over cell-based therapies since exosomes overcome challenges related to transplanting live, proliferative cells, which cannot be entirely controlled in vivo. Immune compatibility, tumorigenicity, embolism, and infection transmission can also be inhibited. The formulation may be assessed for safety, dose, and potency similar to traditional pharmaceutical agents, and hazardous cryo-preservatives can be avoided [21]. Several in vitro and in vivo studies reported utilizing MSC-derived exosomes (cell-free) in regenerative dentistry for periodontal ligament regeneration, oral mucosa healing, pulp regeneration, and bone remodeling in orthodontics [22,23,24,25].

Isolation, purification, and characterization of exosomes are the primary obstacles to consider. Strict GMP procedures and quality control are crucial for creating exosomes of clinical grade. For constant exosome quality and yield, cell source and state, including microenvironmental circumstances, must be maintained uniformly [26]. According to recent in vitro and animal-based studies, MSC-derived exosomes are considered a potential candidate for cell-free regenerative treatment. However, optimized, reproducible, and predictable in vitro or ex vivo models are needed to forecast the efficacy and safety of cell-free-based treatment with MSC-derived exosomes. Future research should also focus on the translation of their properties into clinical and biological uses [19].

## 4. The Application of MSCs in Dentofacial Anomalies

**Figure 1:**
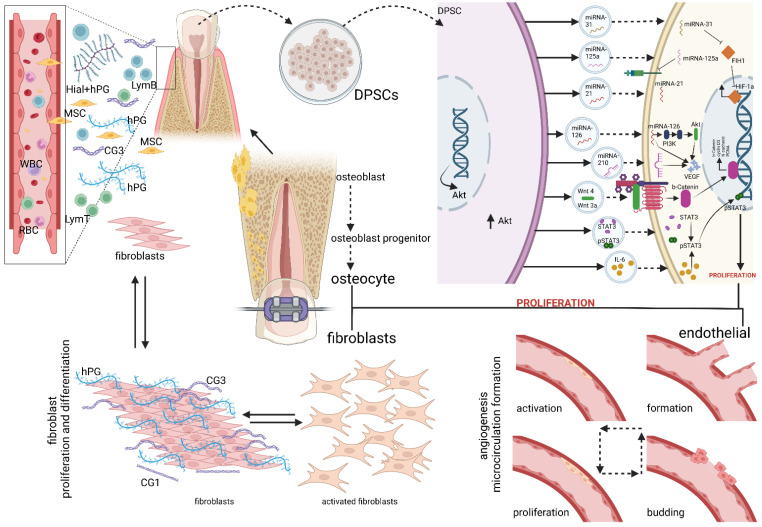
The complex physiological/biological interactions and cellular pathways enabling the clinical application of SCs in orthodontics and dentofacial orthopedics (created using BioRender platform). WBC-white blood cells, RBC: red blood cells, LymB: lymphocyte B, LymT: lymphocyte T, MSC: mesenchymal stem cells, DPSCs: dental pulp stem cells, Hial + hPG: hyaluronic acid + human proteoglycan, CG3: collagen type III, CG1: collagen type I, Wnt 4: protein Wnt 4, HiF-1a: hypoxia-inducible factor, Akt: protein kinase B, PI3K: phosphoinositide 3-kinase, STAT3: signal transducer and activator of transcription 3, IL-6—interleukin 6, VEGF: vascular endothelial growth factor, FIH 1a: factor inhibiting hypoxia-inducible factor 1, mRNA: messenger RNA [47,48].

Complex congenital and developmental craniofacial anomalies can be successfully managed using surgical approaches, involving combinations of allogenic, autogenous, or prosthetic materials to achieve a controlled bone reconstruction [27,39]. These techniques are associated with a risk of side effects and complications such as postoperative pain and infection, resorption of grafted bone, graft versus host disease, and immunosuppression. They often involve bone harvesting from an additional surgical site, resulting in increased patient morbidity [40,41]. Patients with cleft lip and palate, severe facial injuries, and those who had extensive oncological surgery within the head and neck area, mostly require alveolar bone grafts, commonly harvested from the anterior iliac crest. To overcome these limitations, cell-based regenerative therapies are being investigated in craniofacial tissue reconstruction [9,11,13,42,43]. Recently, MSCs have been reported to have the ability to form new bone and regenerate the alveolar cleft [27] (Figure 1). In addition, as stem cells have the ability to differentiate into osteogenic and chondrogenic cells, they have the potential to be used for the individualized repair of temporomandibular joint (TMJ) defects caused by injury, hereditary malformations, or inflammatory processes (arthritis), preserving the mandible in the correct position [44]. While animal studies are progressing to engineer TMJ dysfunction, no clinical human studies evaluating SCs regeneration ability have been conducted yet. In addition, the immunomodulatory effects of MSCs and the bi-directional cross-talk between bone and immune cells in bone regeneration have been the focus of growing research [36,45]. The paracrine effects of macrophages on MSCs and macrophage-mediated regulation of osseous bone regeneration and its impairment have been identified [46].

## 5. The Potential Utilization of Stem Cell-Based Innovations in Clinical and Research Orthodontics

**Figure 2:**
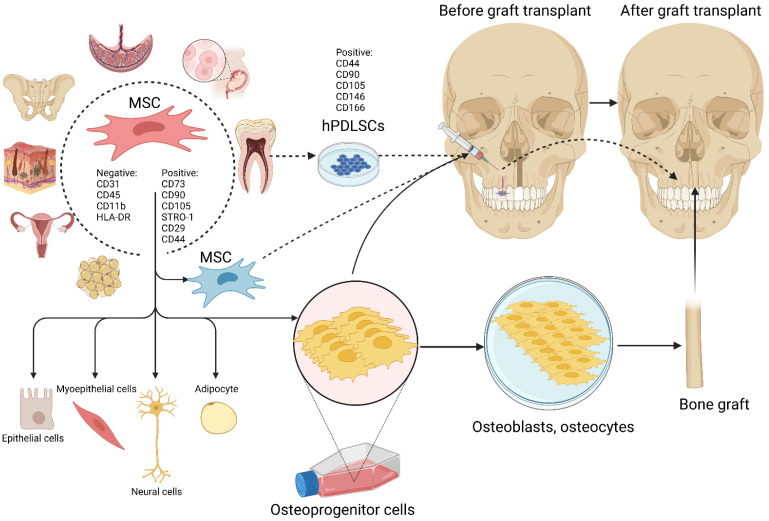
The potential application of SCs in regenerative therapies applicable and designed for orthognatic surgery, including cleft palate. MSC: mesenchymal stem cells, CD: cluster of differentiation, HLA-DR: human leukocyte antigen receptor, STRO-1: mesenchymal precursor cell marker antibody, hPDLSCS: human periodontal ligament stem cells.

Despite a vast range of potential applications of MSCs in orthodontics, as well as DFO, the clinical use of novel modalities in the future may be restricted as a result of a lack of evidence from trials-based results, ethical concerns, and technical limitations. The wide range of hypothetical applications of SCs in orthodontics includes alveolar bone augmentation, enhancement of orthodontic tooth movement, prevention of external root resorption, and periodontal regeneration individualized therapy (Figure 2). The use of gene therapy and molecular biology aspect of orthodontic tooth movement has also been highlighted as regenerative innovations yielded novel DFO alternative treatment options [49].

### 5.1. Alveolar Bone Augmentation Enabling Induction and Acceleration of Orthodontic Tooth Movement

A natural tooth’s (micro)movement is limited by several factors, including soft tissue factors (neuromuscular forces and lip-tooth relationships) and the anatomy of the alveolar bone [50,51]. If tooth movement exceeds the anteroposterior, vertical, and transverse limits of the supporting alveolar bone, then side effects such as dehiscence and gingival recession may develop. In patients with a class III orthodontic discrepancy and “prominent chin”, areas such as the lower incisors, in which buccal and lingual bony support are thin, are at an increased risk of developing dehiscence and fenestrations [52].

While SCs have the potential to generate different tissues, including bone, they can be considered a novel approach for alveolar bone regeneration [53,54]. Clinical trials and systematic reviews reported that SC therapy could be applied for bone ridge augmentation [55], enhancing alveolar bone formation [56], thus expanding the limitations of alveolar bone boundaries and increasing the range of tooth movement.

Orthodontic tooth movement (OTM), a fundamental part of orthodontic treatment, is achieved by alveolar bone and periodontal ligament remodeling in response to mechanical loading [57]. An inflammatory event within the periodontal ligament results in bone resorption in the compression site and bone deposition in the tension site. In the compression region, focal necrosis is followed by osteoclast (derived from hematopoietic SCs) recruitment from the adjacent marrow spaces [58,59]. In fact, SCs provide progenitor cells that could potentially be used to accelerate tooth movement.

Different approaches have been proposed to accelerate OTM and shorten the treatment duration. The risk of adverse effects associated with OTM such as pain, caries, periodontal disease, and root resorption must be considered when developing new orthodontic therapies [60]. Traditionally, invasive surgical techniques (corticotomy and micro-osteoperforation) and also non-surgical methods, including low-intensity laser application, resonance vibration, and local or systemic administration of chemical substances such as prostaglandin, 1,25-dihydroxyvitamin D3, and osteocalcin [61,62,63], were suggested to accelerate and enhance predictable OTM. Recently, in an animal study on a rodent model using CD90 as a marker of periodontal ligament SCs (PDLSCs), it has been demonstrated that the cell numbers increased in the compression site while collagen-1 expression decreased. Conversely, when the orthodontic force was withdrawn, PDLSCs accumulated in the region, and collagen-1 expression increased [64]. These findings suggest that PDLSC function could be intentionally altered to accelerate OTM or stabilize tooth position.

Over the last decade, there has been an increasing interest in utilizing exosomes from ostheotic cells to modify bone remodeling occurring in OTM [65]. Moreover, biological alternatives and novel solutions for orthodontic retention have been tested. Various pharmacological agents have been tested in animal studies to investigate the feasibility and efficacy of novel approaches to reduce relapse without the need for long-term use of retainers [52,66]. Further research into biological factors and pathways involved in tooth movement may improve patients’ compliance and satisfaction in those who complete orthodontic treatment.

### 5.2. SCs in Management of External Root Resorption

External root resorption (ERR) is a common and unfavorable iatrogenic consequence of orthodontic tooth movement, which results in irreversible loss of root dentin and cementum. Several factors are deemed involved in ERR induction, such as individual biological variation, age, sex, genetics, and orthodontic force amount and duration [67,68]. There is a lack of reliable evidence and guidance for clinicians on the effectiveness of interventions used to manage ERR [69]. De novo cementogenesis has been investigated as a treatment modality by Shinagawa-Ohama et al., who reported cementogenic properties of PDL-derived cells in vitro and observed the formation of cellular cementum-like hard tissue in vivo following transplantation of PDL-derived stem and progenitor cells isolated using the outgrowth method [70]. Interestingly, the partial regeneration of certain parts of the damaged teeth, such as dentin and cementum, might be achievable in the future to retain the function and structure of human teeth’ hard tissues. Remarkably, preliminary experiments reported that a whole tooth structure can be bioengineered and transplanted in rodents and dogs [71,72].

### 5.3. The Enhanced Regeneration of Periodontal Structures during Orthodontic Treatment

The potential use of SCs in orthodontics can facilitate a favorable outcome and reduce the risk of side effects associated with fixed orthodontic appliances (FOAs). The traditional orthodontic treatment using FOAs may potentially lead to complications associated predominantly with periodontal structures. A wide range of FOA-related side effects was observed, ranging from gingivitis to periodontitis, fenestration, dehiscence of crestal alveolar bone, gingival recession, and “black triangles effects” [67,68]. Currently, evidence-based and approved treatment modalities include the surgical approach, guided tissue regeneration, bone augmentation, and the application of growth factors and bioactive molecules to induce a physiological process of regeneration if required [73].

Reportedly, the application of SC-targeted periodontal tissue has been recently described to support the FOAs procedure. A study on a murine model with a periodontal fenestration defect and pluripotent SCs implanted with a silk fibroin scaffold in combination with an enamel matrix showed a significantly higher rate of cementum and bone formation [74]. It has been demonstrated that PDLSCs transplanted into a periodontal defect in rats enhanced periodontal regeneration by suppressing the inflammatory response [75]. Additionally, in vivo incubation of induced PDLSCs with dentin non-collagenous proteins resulted in cementum-like tissues along with root dentin surface, increased matrix mineralization, enhanced alkaline phosphatase activity, and upregulated mineralization-associated genes [76]. Furthermore, PDLSCs implanted into the periodontal defect via collagen sponges in periodontal defects of immune-deficient nude rats induce periodontal ligament-like tissue and collagen fiber formation [77]. Significant periodontal tissue regeneration was achieved when a PDLSC sheet was transferred to a miniature pig periodontitis model [78]. As a result, considering the cost aspect, PDLSCs are an ideal source of cells for periodontal regenerative therapies involving alveolar bone structures. Their application may enable more predictable outcomes in complex therapeutic protocols involving alveolar surgeries.

## 6. The Role of Growth Factors in Regenerative Dentofacial Orthopaedics

**Figure 3:**
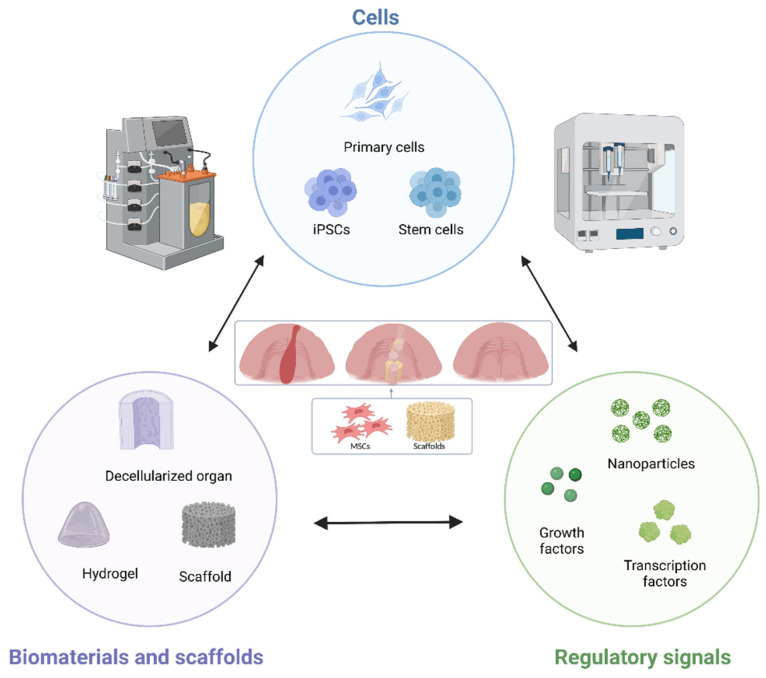
The essential components of tissue engineering in dentofacial malformations (cleft palate, hemifacial microsomia); stem cells, scaffolds, and regulatory signals, including GFs.

Growth factors (GFs), various regulatory endogenic proteins, can enhance the regenerative potential of DFO as an essential component regulating transplanted cells’ interactions and their affinity toward scaffolds, designed for DFO procedures (Figure 3). GFs regulate cell differentiation, migration, survival/apoptosis, and stimulate cell division through mitogenic pathways, affecting a wide range of functions, including tissue repair and regeneration processes [7,12,13,14,79,80,81,82]. They play a pivotal role in tissue repair and regeneration, acting as signaling molecules that modulate cells’ behavior by enabling intracellular communication.

GFs control biological processes during regeneration stages, including differentiation (tumor necrosis factor beta1 (TNF-beta1), platelet-derived growth factor (PDGF), fibroblast growth factor (FGF2), bone morphogenetic proteins 2, 4, 7, and 11 (BMPs), insulin-like growth factor (IGF), nerve growth factor (NGF)), proliferation (PDGF, FGF2, IGF, TGF beta1, stroma cell-derived factor1 (SDF-1), vascular endothelial growth factor (VEGF)), angiogenesis (FGF2, PDGF, VEGF, NGF), neuronal growth (NGF, and chemotaxis (PDGF, FGF2, TGFbeta1, SDF-1) [79,80,81,82,83,84]. Especially, selected human recombinant BMPs, such as BMP2, BMP4, and BMP7, possess substantial osteoconductive effects, inducing mineralization and differentiation of SCs into osteoblasts [80,84]. They can be administered during therapeutic procedures, stimulating alveolar bone regeneration and augmenting with, e.g., biomimetic scaffolds [85]. The investigation of BMPs’ multi-directional biological effects in tissue bioengineering, linking SCs and natural intercellular processes, is pivotal for the further advancement of DFO. Noteworthy, the exact role of these GFs in dentofacial regeneration strategies is still being studied and has not been fully understood.

## 7. Constraints and Pitfalls of RDM

As the new concept of RDM applied in orthodontics and DFO has been primarily demonstrated in pre-clinical and in vitro studies, robust, well-designed trials, observational studies, and in vitro assessments are required to validate the effectiveness and safety of different novel modalities in the dentofacial orthognathics and orthodontics fields. Equally, the cost-benefit ratio of the application of brand-new biotechnological achievements will provide a health-related economic justification for the use of tissue scaffolds, SCs, and growth factors in clinical dental practice. Although the RDM methods have a vast potential for improving patients’ quality of life and treatment outcomes, their overall impact on the management of the global burden of oro-facial diseases is currently deemed limited. Long-term, adequate storage of SCs using innovative cryopreservation methods is necessary to maintain cell viability, their original multidifferentiation potential, and equally phenotypic stability.

Moreover, regulatory bodies must approve the use of SC- and GF-based invasive procedures due to ethical concerns associated with the origin of stem cells. Lastly, while the SCs’ biotechnology requires strict, advanced laboratory protocols and a highly qualified team, there are well-recognized restrictions related to fit-for-purpose facilities and laboratory equipment. Lastly, patients as recipients of early-stage clinical modalities classified as bioengineered methods should be aware of potential complications and the short- and long-term risks involved in order to provide valid, informed consent for often highly experimental and unproven therapies in situations where the expected outcome outweighs the potential risks. What is more, during the consent process, the origin of obtained non-autologous components in RDM must be clearly disclosed.

## 8. Future Implications and Prospects

The application of regenerative approaches combined with 3D computed tomography-based and artificial intelligence technologies is deemed to be the future of routine orthodontics, maxillofacial, and orthognathic surgeries. Inevitably, individualized point-of-care bioengineering utilizing novel biological therapies would provide a breakthrough step for complex craniofacial disorders, traumatic injuries, oncological surgeries, and congenital malformations. The crucial components of tissue bioengineering in dentofacial orthopedic therapies are presented in Figure 3. Healthcare providers and policymakers are obliged to provide optimal strategies, continuously improving therapeutic outcomes. Simultaneously, the international scientific societies should promote state-of-the-art bioengineering applicable to the maxillofacial area with multilevel support from various organizations responsible for regenerative medicine research project funding and acceleration. A worldwide network of laboratories and clinics specializing in bioengineering, underpinned by adequate financial sources, seems essential to pursue innovative RDM modalities, with the prospect of developing point-of-care tissue bioengineering, utilizing affordable bioreactors and know-how assets. Expectedly, SCs-based novel regenerative therapies can support the treatment of TMJ disorders, particularly as a result of pathological degeneration and/or inflammatory processes, including osteoarthritis, impacting TMJ structure and function [44,86]. These therapies encompass a revolutionary array of predictable corrections for TMJ-related malfunctions associated with articulation and occlusion.

As future research projects within the RDM/DFO fields require complex, multifaceted, and sophisticated strategies, the utilization of machine learning to support dedicated biologically active molecules and the design of optimal SCs–biomaterials–signaling transmitters are deemed essential components of dynamically evolving bioengineering technology. Their common primary aim should be focusing on the delivery of smart, patient-centered regenerative modalities widely applicable in interventional orthodontics and translational medicine.

## 9. Limitations of Scoping Review

While a standardized systematic review approach has not been applied due to the presented reasons, the search outcome could be potentially compromised if some evidence is accidentally omitted. Secondly, as quality assessment and data synthesis, including publication bias evaluations, were not conducted, the scientific soundness of the presented evidence cannot be verified. Regarding English language restrictions during data search, this might potentially affect the search outcome, including the risk of missing relevant evidence [87]. On the other hand, Morrison et al. [88] found no evidence of systematic bias from the use of language limits. Arguably, the utilized simplified scoping review strategy could enhance search outcomes by narrowing exclusion criteria and including existing results obtained from cumulative systematic reviews, umbrella reviews, clinical trials, and preliminary reports, which are typically excluded in structured, robust protocols of systematic reviews. Primarily, the significant heterogeneity of existing evidence associated with orthodontic-related regenerative therapy prevented the use of a comprehensive systematic review and meta-analysis/data synthesis. Expectedly, this perspective review will deliver an impetus for further robust research, adding value to the regenerative medicine field and clarifying the scientific soundness of DFO evidence.

## 10. Conclusions

While adult MSC-like cells originating from the maxillofacial region have multi-differential potential and are an accessible source of SCs, reportedly they have the potential to be used in specifically designed, highly individualized regenerative therapies in orthodontics and orthognathic surgery. Based on current data from in vitro and animal pre-clinical studies, the application of combined cell-based and cell-free strategies might optimize treatment outcomes and equally reduce treatment duration. Undoubtedly, obvious challenges exist associated with treatment modalities, including the specific characterization and incomplete understanding of stem cell/progenitors’ behavior in vivo, specifically in guided regeneration processes of the complex craniofacial tissues, originating from three different primary germ layers. The broader implementation of standardized cell culture protocols and efficient and scalable production of cell-derived products is deemed a prerequisite for clinical translation. Adequate financial support for ambitious multidisciplinary projects in regenerative biomedicine, funded by the European Research Council, the National Institute of Health, and national/regional research bodies, extending beyond existing knowledge, is required to validate the practical benefits of such interventions for clinicians aiding in providing comprehensive, advanced orthodontics involving precise control of hard/soft tissue modifications.

## Figures and Tables

**Table 1:** The characteristics and selected primary results of in vitro and in vivo studies investigating stem cells (oral and non-oral-derived) reflecting their potential use in regenerative dentofacial orthopedics.

Study	Year	Study Design	Type of Stem Cells Used	Primary Aim	The Main Outcome
Ma et al.	2008	In vitro and in vivo	Human periodontal ligament stem cells (HPDLSCs)	To investigate the biologic effect of dentin non-collagenous proteins on HPDLSCs	HPDLSCs enhance alkaline phosphatase activity, increase matrix mineralization, and upregulate expression of mineralization-associated genes
Ding et al.	2010	Experimental animal	Periodontal ligaments stem cells (PDLSCs)	The use of PDLSCs sheet to cure periodontitis	PDLSCs possess low immunogenicity and marked immunosuppression via PGE2-induced T-cell anergy
Grimm et al.	2011	Experimental animal	PDLSCs	To investigate the capability of PDLSCs to differentiate into osteogenic lineage	PDLSCs are capable of regenerating elements of bone and collage fibers
Rickert et al.	2011	(Human) randomized clinical trial	Mesenchymal stem cells seeded on bone mineral (MSCs)	To assess bone formation after maxillary sinus lift using mesenchymal stem cells	MSCs can induce bone formation
Duan et al.	2011	In vitro	Induced pluripotent stem (iPS)	To investigate the capabilities and advantages of periodontal tissue regeneration using iPS	iPS cells combined with enamel matrix derivatives promote the formation of new cementum, alveolar bone and periodontal ligament
Yamada et al.	2013	(Human) cohort	Bone marrow-derived mesenchymal stem cells	The assessment of bone formation using tissue-engineered bone in cases of severe maxillary bone resorption	Tissue-engineered bone can regenerate bone formation
Feng et al.	2016	Experimental animal	PDL stem/progenitor cells	The role of PDL stem cells in PDL remodeling after mechanical force application	PDL stem cells can respond to mechanical force and are required for PDL recovery
Shinagawa-Ohama et al.	2017	In vitro and in vivo	Dental follicle and PDL-derived MSCs	To investigate cementogenic potential of dental follicle and PDL-derived MSCs	PDL stem cells potentially facilitate the de novo cellular cementogenesis
Nagata et al.	2017	Experimental animal	PDL stem cells (PDLSCs)	To investigate the regenerative potential of PDLSCs in tooth-supporting tissues	PDLSCs enhance periodontal regeneration by suppressing response via TNF-α production

**Table 2:** Types of stem cells (DSCs) originating from the oro-facial and maxillofacial area. Origin (primary isolation), biological properties, and potential application in regenerative dentofacial orthopedics.

Type of DSCs	Origin	Properties and Potential Applications
Dental pulp stem cells (DPSCs) 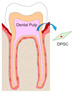	Isolated from the pulp tissue of extracted human teeth, mainly third molars	Confirmed potential for clinical application in various systemic diseases by differentiating into neurons, osteoblasts, liver cells, and β cells of the islet of the pancreas, as well as: human exfoliated deciduous teeth, apical papilla, periodontal ligament, dental follicle tissue
Dental follicle progenitor cells (DFPCs) 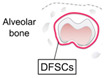	Isolated from the loose mesenchymal tissue surrounding the developing tooth germ (dental follicle)	Capable of multipotent differentiation with high pluripotency DFPCs can differentiate into osteoblasts, adipocytes, chondrocytes, cementoblasts and periodontal ligament cells, as well as neuronal cells
Stem cells from apical papilla (SCAP) 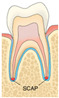	Present in the apical papilla of permanent immature teeth	Adult stem cells that have variable functions, including: reduction in inflammation, diminishment of scarring (fibrosis process), improvement of immune stability Broad potential for application in regenerative medicine, particularly maxillofacial surgery. Significant proliferative capacity compared to pulp-originating SCs (2–3× greater)
Periodontal ligament stem cells (PDLSCs) 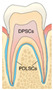	Present in the perivascular space of the periodontium	Responsible for the regeneration of periodontal components: the periodontal ligaments, alveolar bone, and cementum
Stem cells from human exfoliated deciduous teeth (SHED) 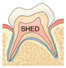	Present within exfoliated deciduous tooth pulp tissue	Ability to differentiate into a broad range of various cell types, such as osteoblasts, adipocytes, and neurons

## Data Availability

Not applicable.
